# The Effectiveness of Short Message Service to Assure the Preparation-to-Colonoscopy Interval before Bowel Preparation for Colonoscopy

**DOI:** 10.1155/2015/628049

**Published:** 2015-02-22

**Authors:** Jongha Park, Tae-Oh Kim, Nae-Young Lee, Hyoungjun Kim, Eun Hee Seo, Nae-Yun Heo, Seung Ha Park, Young-Soo Moon

**Affiliations:** ^1^Division of Gastroenterology, Department of Internal Medicine, Haeundae Paik Hospital, College of Medicine, Inje University, 1435 Jwa-dong, Haeundae-gu, Busan 612-896, Republic of Korea; ^2^Department of Nursing, Silla University, Busan, Republic of Korea

## Abstract

*Background/Aims.* The preparation-to-colonoscopy (PC) interval is one of several important factors for the bowel preparation. Short message service (SMS) reminder from a cellular phone has been suggested to improve compliance in various medical situations. We evaluated the effectiveness of SMS reminders to assure the PC interval for colonoscopy. *Methodology.* This prospective randomized study was investigator blinded. In the No-SMS group, patients took the first 2 L polyethylene glycol (PEG) between 6 and 8 PM on the day before colonoscopy and the second 2 L PEG approximately 6 hours before the colonoscopy without SMS. In the SMS group, patients took first 2 L PEG in the same manner as the No-SMS group and the second 2 L PEG after receiving an SMS 6 hours before the colonoscopy. *Results.* The SMS group had a lower score than the No-SMS group, according to the Ottawa Bowel Preparation Scale (*P* < 0.001). Multivariate logistic regression analysis showed that compliance with diet instructions (odds ratio (OR) 2.109; 95% confidence interval (CI), 1.11–3.99, *P* = 0.022) and intervention using SMS ((OR) 2.329; 95% (CI), 1.34–4.02, *P* = 0.002) were the independent significant factors for satisfactory bowel preparation. *Conclusions.* An SMS reminder to assure PC interval improved the bowel preparation quality for colonoscopy with bowel preparation.

## 1. Introduction

Adequate bowel preparation isessential for a successful colonoscopic examination. Inadequate bowel preparation may lead to a lower cecal intubation rate, a prolonged examination time, a decreased lesion identification rate, an increased complication rate, and the increased discomfort of the examinee as well as the examiner [1–4]. The following factors have been reported as predictors of inadequate bowel preparation for colonoscopy: older age, female sex, diabetes mellitus, constipation, history of abdominal or pelvic surgery, compliance with preparation instructions, and bowel preparation type [5, 6].

Some previous studies have reported that inadequate bowel preparation and lower cecal intubation rate are more frequent in afternoon colonoscopy than in morning colonoscopy [7–9]. However, recent studies have shown that the time interval between bowel preparation and the start of colonoscopy is a more significant factor than the timing of the colonoscopy [10, 11]. Furthermore, we recently completed a prospective observational study examining the optimal preparation-to-colonoscopy (PC) interval, which means the time interval between the completion of the last polyethylene glycol (PEG) ingestion and the colonoscopy starting time, and the results showed that colonoscopies with a PC interval of 3 to 5 hours had the best bowel preparation quality score in split-dose PEG bowel preparation [12].

There are many different protocols for bowel preparation and the preferences to a certain bowel preparation protocol differ between countries by their medical environment. In addition, different types of daily diet may affect the quality of bowel preparation. Because the past medical history and types of daily diet cannot be easily modifiable, improving the compliance with the appropriate preparation instructions can be one of the best ways to increase the bowel preparation quality.

According to a recent report, the number of worldwide mobile cellular subscribers reached the 4-billion mark in late 2008 and continues to increase [13]. In addition, several studies showed that the short message service (SMS) reminder was associated with a decrease in nonattendance [14–17]. Because bowel preparation with a split-dose PEG is obviously uncomfortable, patients report difficulty complying with the suggested timing of PEG ingestion. Therefore, we applied SMS to assure an appropriate PC interval and to improve the bowel preparation quality in this study. Therefore, we conducted a prospective randomized controlled study to evaluate the effectiveness of using short message service (SMS) from a mobile phone to increase patient compliance with the suggested PC interval for satisfactory bowel preparation for afternoon colonoscopy [14–18].

## 2. Materials and Methods

### 2.1. Subjects

We prospectively enrolled consecutive outpatients, aged 18 and 80 years, who were scheduled for an afternoon colonoscopy. This study was conducted at a single university hospital between October 2011 and April 2012. Patients with one or more of the following conditions were excluded: unavailability of a mobile phone or SMS, age younger than 18 years, pregnancy, breastfeeding, history of large-bowel resection, renal failure (serum creatinine ≥ 3.0 mg/dL (normal 0.8–1.4)), drug addiction, major psychiatric illness, allergy to PEG, and refusal to participate in the study. All patients provided written informed consent. This study was approved by the institutional review board and registered in the clinical trial database at ClinicalTrials.gov NCT01675739.

### 2.2. Randomization and Blinding

We generated a randomization schedule using randomly computed blocks, according to the website (http://www.randomization.com/). An investigator separate from the colonoscopy procedures randomly assigned patients to the No-SMS group or the SMS group, according to the schedule. The colonoscopists and nurses who scored the bowel preparation and recorded the colonoscopic data were blind to the randomization results during the study period. A separate investigator managed the data and performed the statistical analysis.

### 2.3. Bowel Preparation Protocol and Scheduled SMS

All patients were instructed to start a low-fiber diet 3 days before the colonoscopy and received a list of unacceptable foods. On the day before the colonoscopy, patients followed a regular diet for breakfast and lunch and then a soft diet for dinner. Only clear liquids were allowed up until 2 hours before the procedure on the day of the colonoscopy. All patients received a printed bowel preparation manual and the reservation time and date of their colonoscopy by the document. Patients in the No-SMS group took the first 2 L of PEG solution (Colyte, Taejoon Pharm Inc., Seoul, Republic of Korea; 236 g PEG, 22.74 g Na_2_SO_4_, 6.74 g NaHCO_3_, 5.86 g NaCl, and 2.97 g KCL) between 6 and 8 PM on the day before the colonoscopy and the second 2 L of PEG approximately 6 hours before the colonoscopy. Patients in the SMS group took the first 2 L of PEG in the same manner as the No-SMS group and the second 2 L of PEG approximately 6 hours before the colonoscopy, after receiving a scheduled SMS (*Please begin taking the second dose of the prepared solution now.*) as notified at the time of the randomization step. We sent the computer-generated programmed text message (SMS) to the SMS group using the existing SMS reminder system for outpatient appointment in our hospital. Patients were instructed to drink 250 mL of the PEG every 10 minutes.

### 2.4. Data Collection

All patients were given a questionnaire to assess the timing of the last PEG dose, the amount of preparation taken, and the level of compliance with the instructions for bowel preparation and diet. Taking at least 75% of the preparation volume was regarded as appropriate for bowel preparation. To assess the compliance of patients, patients rated their compliance with the received instructions and diet.

The following data were collected from each patient: age, gender, body mass index, the status of intervention using SMS, history of colonoscopy, history of abdominal or pelvic surgery, familial history of colon cancer, the reason for the colonoscopy, chronic comorbidities (diabetes, hypertension, stroke, and thyroid disease), and PC interval.

### 2.5. Evaluation of the Bowel Preparation Quality and Colonoscopic Procedure

The Ottawa Bowel Preparation Scale was used to evaluate the bowel cleansing (Table 1) [19]. The three endoscopists participating in this study were instructed on the assessment of bowel preparation quality according to the Ottawa Scale, and each performed calibration exercises involving 20 colonoscopies before starting this study. To determine intraobserver agreement, the value was calculated on a per-patient basis; the results were very positive (*κ* = 0.89). The *κ* interclass correlation coefficient was then calculated to evaluate the interrater reliability of the bowel preparation quality ratings for the 3 raters. The *κ* interclass correlation coefficient was 0.87, indicating a high level of interrater consistency. All study procedures were performed by 3 highly experienced colonoscopists (Tae-Oh Kim, Jongha Park, and Eun Hee Seo), each having performed more than 10,000 colonoscopies. An afternoon colonoscopy was defined as a procedure starting from 1:30 PM on. We tried to keep the reservation time for study patients.

### 2.6. Statistical Analysis

The sample size was based on the comparison of bowel preparation quality using the Ottawa Bowel Preparation Scale between the No-SMS group and the SMS group. We hypothesized that the SMS group would show a 20% improved Ottawa Bowel Preparation score over the No-SMS group. With a study power of 90%, a confidence interval of 95%, and an alpha-error of 5%, the sample size was estimated by the Power Analysis and Sample Size Software program to be 130 patients in each group. Thus, we planned to enroll a total of 280 patients with a built-in dropout rate of approximately 10%.

All statistical analyses were performed with SPSS version 12.0K (SPSS Inc., Chicago, IL, USA). Continuous variables were reported as mean ± standard deviations and categorical variables as percentages. Associations between categorical variables were evaluated by Pearson's chi-squared test and Fisher's exact test and continuous variables by Student's *t*-test. Those factors that were statistically significant (*P* < 0.05) in univariate analysis were included in multivariate analysis. Multivariate logistic regression analysis was used to assess the factors affecting the bowel preparation quality.

## 3. Results

### 3.1. Baseline Characteristics

**Figure 1:**
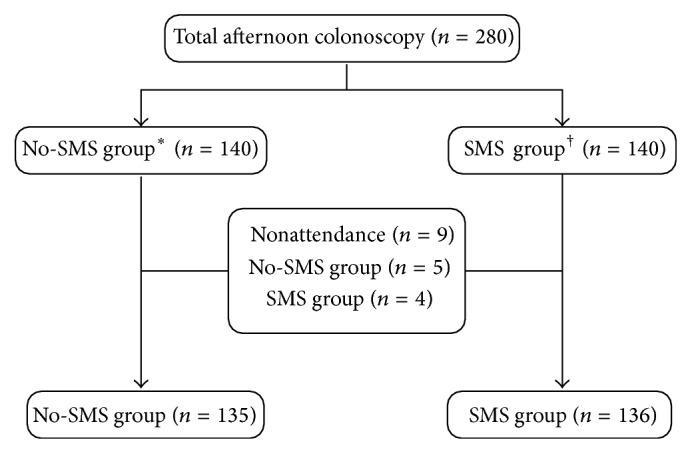
Flow of patients through the study. In the No-SMS group ^*^, patients took the first 2 L of PEG solution between 6 and 8 PM on the day before colonoscopy and the second 2 L of PEG approximately 6 hours before the colonoscopy with no SMS reminder. In the SMS group ^†^, patients took first 2 L of PEG in the same manner as the No-SMS group and the second dose of PEG 2 L after receiving an SMS 6 hours before an afternoon colonoscopy. PEG, polyethylene glycol; SMS, short message service of cellular phone.

A total of 280 consecutive patients were enrolled in this study. Nine patients were excluded due to nonattendance; therefore, 135 patients of the No-SMS group and 136 patients of the SMS group were evaluated (Figure 1, Table 2). The patient population consisted of 46.5% men, and the mean age was 54.7 years (range, 20–80). Of these, 116 patients (42.8%) had previous abdominal or pelvic surgery, and 136 patients (50.2%) had undergone a colonoscopy previously. Five (1.8%) patients failed to ingest more than 75% of the PEG solution, and those patients were all in the No-SMS group. Two hundred twenty-eight (84.1%) patients complied with diet instructions, 80% in the No-SMS group and 88.2% in the SMS group. The indications for colonoscopy were screening (52.8%), symptoms (32.5%), and surveillance (14.8%). Symptoms leading to the colonoscopy included rectal bleeding, anemia, positive stool occult blood, significant weight loss, abdominal pain, discomfort, bloating, changes in bowel habits, constipation, and diarrhea. There were no differences in the age, gender, history of colonoscopy, familial history of colorectal cancer, or comorbid diseases between the No-SMS and the SMS groups. The No-SMS group presented with more symptoms of rectal bleeding, changes in bowel habits, and abdominal pain/discomfort/bloating than the SMS group. The SMS group had a history of abdominal or pelvic surgery and anemia more frequently than the No-SMS group. Cecal intubation was attained successfully in all cases except for one member of the SMS group. There was no significant difference of polyp detection rate, adenoma detection rate, and colonoscopy withdrawal time between No-SMS group and SMS group. We confirmed that all patients of the SMS group had received the proper SMS before taking the second dose PEG.

### 3.2. The Association of Bowel Preparation Quality with the PC Interval and Intervention Using SMS

**Figure 2:**
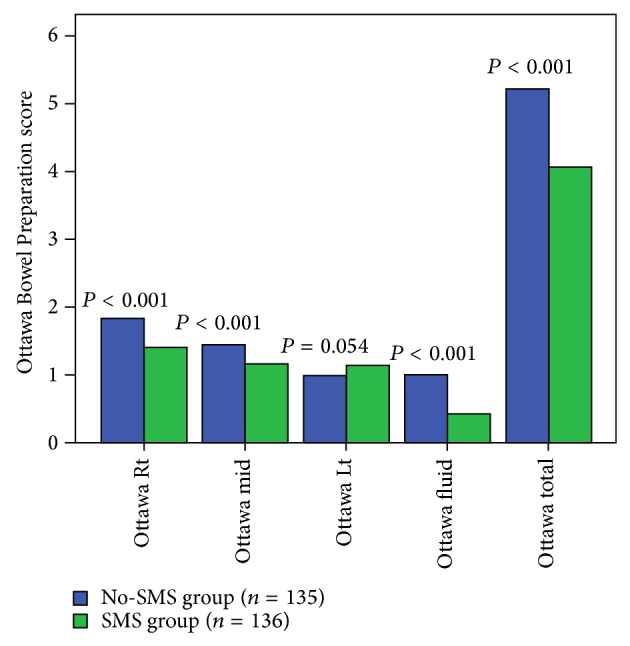
The association of bowel preparation quality with the PC interval and intervention using SMS. Ottawa Rt is Ottawa score of the right colon (cecum, ascending colon). Ottawa mid is Ottawa score of the midcolon (transverse, descending colon). Ottawa Lt is Ottawa score of the left colon (the rectosigmoid colon). Ottawa fluid is the fluid quantity of the entire colon. PC interval, preparation-to-colonoscopy interval; SMS, short message service of cellular phone.

The median total score of the Ottawa Scale for the total 271 patients was 4 (range: 0–12). The total score of the Ottawa Scale for the SMS group (median 3, range 0–12) was lower than the score for the No-SMS group (median 5, range 1–9) (*P* < 0.001). In the analyses of each segment of the colon and the fluid quantity, the Ottawa score tended to decline from the right colon to the left colon. The scores for each segment were lower in the SMS group than in the No-SMS group (*P* < 0.001) for all segments except the left colon (*P* = 0.054). The score for fluid collection was lower in the SMS group than in the No-SMS group (*P* < 0.001) (Figure 2).

### 3.3. The Relationship between Intervention Using SMS and the PC Interval

We observed that the bowel preparations scoring between 0 and 5 on the Ottawa Scale resulted in only liquid material without stool in all colon segments. Therefore, we determined that an Ottawa Scale score of 5 or less was acceptable for detecting flat lesions during the colonoscopy. Thus, we set a total Ottawa Scale score of 5 as the cutoff level for satisfactory bowel preparation after discussing with the colonoscopists participating in this study. A satisfactory total Ottawa score (≤5) was obtained more frequently in the SMS group (79.4%) than in the No-SMS group (57.8%). In addition, more patients from the SMS group (41.9%) had a PC interval of 3 to 5 hours than those from the No-SMS group (13.3%) (Table 3).

### 3.4. Analyses of Factors Associated with Satisfactory Bowel Preparation

We evaluated some factors that might affect bowel preparation quality. As mentioned above, a satisfactory bowel preparation was determined by an Ottawa Bowel Preparation score of 0 to 5. In univariate analysis, history of abdominal or pelvic surgery (*P* = 0.013), abdominal pain/discomfort/bloating (*P* = 0.001), change in bowel habits (*P* = 0.033), intervention using SMS (*P* < 0.001), compliance with diet instructions (*P* = 0.002), ≥75% of PEG ingested (*P* = 0.001), mean PC interval (*P* < 0.001), and PC interval 3–5 h (*P* = 0.028) were significantly associated with satisfactory bowel preparation (Table 4). Multivariate logistic regression analysis showed that compliance with diet instructions (odds ratio (OR) 2.109; 95% confidence interval (CI), 1.11–3.99, *P* = 0.022) and intervention using SMS ((OR) 2.329; 95% CI, 1.34–4.02, *P* = 0.002) were independent significant factors for satisfactory bowel preparation (Table 5).

## 4. Discussion

Some recent studies have shown that the time interval between bowel preparation and the start of colonoscopy is a more significant factor than the timing of the colonoscopy [10, 11]. However, because bowel preparation with the PEG solution is a very unpleasant experience due to the large volume and poor taste of the PEG solution, many patients find it difficult to take the appropriate volume of PEG solution on time. Education about bowel preparation is considered an important factor to improve bowel preparation quality [12, 20], and Spiegel et al. suggested using booklets about bowel preparation to improve the preparation quality and behavior of patients [21].

Therefore, we intended to evaluate whether a simple SMS reminder could raise patient compliance with a PC interval of 3–5 hours and improve the rate of satisfactory bowel preparation, as classified by an Ottawa Bowel Preparation score of 0–5 [12]. Because we observed that bowel preparation quality that scored between 0 and 5 indicated only liquid without formed stool in any colon segment, we considered an Ottawa Bowel Preparation score of under 5 to be acceptable for detection of flat polyps.

In this study, SMS intervention to assure a PC interval 3–5 hours induced more patients to take the PEG solution within the expected time interval (SMS group versus No-SMS group: 41.9% versus 13.3%). Furthermore, more patients in the SMS group had a satisfactory Ottawa Bowel Preparation score (≤5) than patients in the No-SMS group (79.4% versus 57.8) (Table 3). The Ottawa score of each colon segment and the overall quantity of residual fluid were significantly associated with a lower score in the SMS group, except left side of colon (Figure 2). Recently, the preparation of the right side of colon has been considered important because the detection of flat polyps can be difficult if the right side colon preparation is poor. Thus, a simple SMS reminder for bowel preparation can induce more effective bowel preparation and might increase the polyp detection rate on the right side of the colon.

In multivariate logistic regression analysis, the independent significant factors for a satisfactory Ottawa Bowel Preparation score (≤5) were in compliance with diet instructions and intervention using SMS (Table 5). Compliance with diet instructions has been revealed as a significant contributor to bowel preparation in previous studies [12, 20]. Intervention using SMS was an independent significant factor for satisfactory bowel preparation (Ottawa score 0–5). A PC interval of 3–5 hours was a significant factor in univariate analysis, but it was not an independent factor for satisfactory bowel preparation in multivariate analysis. This finding differs from our previous observational study, which showed that a PC interval of 3–5 hours was an independent significant factor for satisfactory bowel preparation. However, our study results showed that the SMS group more frequently had satisfactory bowel preparation, as well as a PC interval of 3–5 hours. Therefore, we speculate that this inconsistency was caused by the small study size and the different study design, and a larger randomized study is needed.

SMS intervention prompted the ingestion of an appropriate volume of PEG solution. In this study, 5 patients did not ingest more than 75% of the PEG solution, and those patients were all in the No-SMS group. This finding indicates that SMS reminders encouraged patients to ingest an appropriate amount of the PEG solution. That the ingestion of an appropriate volume of PEG was a significant factor for bowel preparation indicates the important effect an SMS reminder may have [12, 20]. Some demographic factors, including age, gender, body mass index (BMI), constipation, diabetes, and previous history of abdominal or pelvic surgery, have been associated with poor bowel preparation quality in previous studies [5, 6, 12, 20]. However, our study's results showed that these factors were not significantly related to satisfactory bowel preparation in multivariate analysis.

The International Telecommunication Union (ITU), which is the UN agency for information and communications technology, reported that the number of worldwide mobile cellular subscribers reached the 4-billion mark in late 2008 and continues to increase. Furthermore, it showed that mobile technology is changing peoples' lives [13]. Thus, several studies concerning the usefulness of an SMS reminder to improve attendance at outpatient appointments showed that the SMS reminder was associated with a decrease in nonattendance [14–17]. Therefore, we applied SMS to assure an appropriate PC interval and to improve the bowel preparation quality in this study.

As our study shows, an SMS reminder was effective in assuring a PC interval of 3–5 hours and led to a more satisfactory bowel preparation quality in the SMS group. Thus, if we use SMS to reinforce diet instructions and timetables for the ingestion of PEG, we could achieve more satisfactory bowel preparation quality in more patients. However, regarding the practical application of SMS reminders, the cost-effectiveness is an important consideration. According to a recent systemic review about use of telephone and SMS reminders to improve attendance at hospital appointments, the mean cost of SMS or automated phone call was $0.17 per case [17]. However, in Republic of Korea, the mean cost for one SMS is only $0.02 (*₩*20) per case. Moreover, the cost of colonoscopy with or without sedation is very different among countries. There is no generally applicable previous study or guideline regarding the cost-effectiveness of an SMS reminder for colonoscopy bowel preparation; thus, the practical application of an SMS reminder for bowel preparation before colonoscopy must consider each country's medical environment. If an SMS reminder is cost-effective, further benefit may be gained from multiple SMS reminders for diet instructions and first and second PEG intake time. Additionally, considering the explosive growth of smartphone use worldwide, a smartphone application for the instructions of bowel preparation procedures could be a more effective modality for satisfactory bowel preparation.

This study has several limitations. First, it was a single tertiary center study; therefore, unexpected confounding factors could have affected the assessment of bowel preparation quality and patient recruitment. Second, we enrolled subjects only from the population of outpatient afternoon colonoscopies. At the time of the design of this study, we wanted to avoid patient discomfort caused by receiving an SMS in the early morning, as the effectiveness of an SMS reminder was unknown. In our country, most of the diagnostic colonoscopies performed are outpatient based; therefore, we enrolled subjects from the population of outpatient colonoscopies.

Additionally, the differences of bowel preparation protocol, communication pattern, and type of daily diet in population across the world may affect bowel preparation quality. Owing to the different medical environment or the lack of infrastructure for mobile phone service, the SMS may not be available in some countries. Thus, it may be difficult to apply the results of this study to the current general clinical environment immediately. Therefore, to confirm the generalizability, we expect further studies using SMS in different populations across the world.

## 5. Conclusions

In conclusion, SMS reminders to assure a PC interval improved bowel preparation quality in afternoon colonoscopies with a split-dose PEG bowel preparation. In the future, further large scale multicenter randomized trials are necessary to evaluate the cost-effectiveness and efficacy of SMS reminders for morning colonoscopies.

## Figures and Tables

**Table 1:** Ottawa Bowel Preparation Quality Scale.

Quality of preparation	Score
Individual evaluation of right, mid, and left colon	
No liquid	0
Minimal liquid, no suctioning required	1
Suction required to see mucosa	2
Wash and suction	3
Solid stool, not washable	4
Evaluation of the entire colon	
Overall quantity of fluid	0–2

Total Ottawa score (0–14) is obtained by adding the scores for individual evaluation of the right, mid, and left colon with the score of overall fluid in the entire colon.

**Table 2:** Basal characteristics of the study population.

Characteristics	No-SMS^*^ group (*n* = 135)	SMS group (*n* = 136)	Total (*n* = 271)	*P* value
Age, y (range)	55.8 ± 12.3 (20–80)	53.7 ± 10.4 (26–77)	54.7 ± 11.4 (20–80)	0.143
Gender (male : female)	63 (46.7) : 72 (53.3)	63 (46.3) : 73 (53.7)	126 (46.5) : 145 (53.5)	0.955
Body mass index, kg/m^2^ (range)	23.2 ± 2.8 (16–32)	23.6 ± 2.6 (16.7–32.9)	23.4 ± 2.7 (16.0–32.9)	0.210
History of colonoscopy	75 (55.6)	61 (44.9)	136 (50.2)	0.078
Abdominal or pelvic surgery	36 (26.7)	80 (58.8)	116 (42.8)	**<0.001**
Familial history of colorectal cancer	8 (5.9)	9 (6.6)	17 (6.3)	0.814
Chronic comorbid disease				
Diabetes	9 (6.7)	6 (4.4)	15 (5.5)	0.417
Thyroid disease	3 (2.2)	6 (4.4)	9 (3.3)	0.315
Indication of colonoscopy				**<0.001**
Screening	53 (39.3)	90 (66.2)	143 (52.8)	
Surveillance	25 (18.5)	15 (11.0)	40 (14.8)	
Symptoms	57 (42.2)	31 (22.8)	88 (32.5)	
Symptoms				
Rectal bleeding	11 (8.1)	1 (0.7)	12 (4.4)	**0.003**
Anemia	1 (0.7)	8 (5.9)	9 (3.3)	**0.018**
Positive stool occult blood	1 (0.7)	0	1 (0.4)	0.315
Significant weight loss	2 (1.5)	0	2 (0.7)	0.154
Abdominal pain/discomfort/bloating	34 (25.2)	14 (10.3)	48 (17.7)	**0.005**
Change in bowel habits	7 (5.2)	0	7 (2.6)	**0.007**
Constipation	2 (1.5)	3 (2.2)	5 (1.8)	0.658
Diarrhea	5 (3.7)	5 (3.7)	10 (3.7)	0.991
Mean PC interval^†^, hour (range)	7:03 ± 1:38 (2:30–13:00)	5:02 ± 2:02 (1:00–9:00)	6:02 ± 2:06 (1:00–13:00)	**<0.001**
Compliance with diet instructions	108 (80)	120 (88.2)	228 (84.1)	0.064
≥75% of PEG^‡^ ingested	130 (96.3)	136 (100)	266 (98.2)	**0.023**
Intubation to cecum	135 (100)	135 (99.3)	270 (99.6)	0.318
Polyp detection rate (%)	37.8	41.2	39.5	0.567
Adenoma detection rate (%)	31.1	30.9	30.1	0.968
Colonoscopy withdrawal time, second (range)	371.3 ± 39.5 (258–491)	379.6 ± 47.7 (258–755)	375.4 ± 44.0 (258–755)	0.119

Values are mean ± standard deviation or number (%).

SMS^*^, short message service of cellular phone; PC interval^†^, preparation-to-colonoscopy interval; PEG^‡^, polyethylene glycol.

**Table 3:** The relationship of intervention using SMS and PC interval ^*^.

	No-SMS^†^ group (*n* = 135)	SMS group (*n* = 136)	Total (*n* = 271)	*P* value
PC interval 3–5 h	18 (13.3)	57 (41.9)	75 (27.7)	<0.001
Satisfactory Ottawa scale (≤5)	78 (57.8)	108 (79.4)	186 (68.6)	<0.001

PC interval^*^, preparation-to-colonoscopy interval; SMS^†^, short message service of cellular phone.

**Table 4:** Univariate analysis of factors associated with satisfactory bowel preparation (Ottawa Scale 0–5).

	Satisfactory preparation (*n* = 186)	Unsatisfactory preparation (*n* = 85)	*P* value
Age, y (range)	53.8 ± 11 (20–77)	56.8 ± 12.1 (25–80)	0.057
Gender (male : female)	82 (44.1) : 104 (55.9)	44 (51.8) : 41 (48.2)	0.240
Intervention using SMS	108 (58.1)	28 (34.6)	**<0.001**
Body mass index, kg/m^2^ (range)	23.5 ± 2.7 (16.0–32.9)	23.1 ± 2.5 (18.4–29.4)	0.175
History of colonoscopy	95 (51.1)	41 (48.2)	0.664
History of abdominal or pelvic surgery	89 (47.8)	27 (31.8)	**0.013**
Familial history of colon cancer	14 (7.5)	3 (3.5)	0.208
Indication of colonoscopy			0.724
Screening	101 (54.3)	42 (49.4)	
Surveillance	27 (14.5)	13 (15.3)	
Symptoms	58 (31.2)	30 (35.3)	
Chronic comorbid disease			
Diabetes	9 (5.9)	6 (7.1)	0.567
Thyroid disease	6 (3.2)	3 (3.5)	0.938
Symptoms			
Rectal bleeding	9 (4.8)	3 (3.5)	0.759
Anemia	8 (4.3)	1 (1.2)	0.281
Positive stool occult blood	1 (0.5)	0 (0.0)	0.513
Significant weight loss	1 (0.5)	1 (1.2)	0.530
Abdominal pain/discomfort/bloating	23 (12.4)	25 (29.4)	**0.001**
Change in bowel habits	2 (1.1)	5 (5.9)	**0.033**
Diarrhea	8 (4.3)	2 (2.4)	0.729
Constipation	3 (1.6)	2 (2.4)	0.650
Polyp detection rate (%)	75 (40.3)	32 (37.6)	0.996
Adenoma detection rate (%)	57 (30.6)	27 (31.8)	0.587
Compliance with diet instructions	165 (88.7)	63 (74.1)	**0.002**
≥75% of PEG^*^ ingested	186 (100)	80 (94.1)	**0.001**
Mean PC interval^†^ (h)	5:42 ± 2:02 (1:00–9:50)	6:47 ± 2:04 (2:30–13:00)	**<0.001**
PC interval 3~5 h	59 (31.7)	16 (18.8)	**0.028**

Values are mean ± standard deviation or number (% or range).

PEG^*^, polyethylene glycol; PC interval^†^, preparation-to-colonoscopy interval.

**Table 5:** Multivariate analysis of factors associated with satisfactory bowel preparation (*N* = 271).

Variable	Odds ratio	[95% CI^*^]	*P* value
Intervention using SMS^†^	2.329	1.34–4.02	0.002
Compliance with diet instructions	2.109	1.11–3.99	0.022

CI^*^, confidence interval; SMS^†^, short message service of cellular phone.
